# Heavenly HELLS? A potential new therapeutic target for retinoblastoma

**DOI:** 10.18632/oncoscience.502

**Published:** 2020-05-01

**Authors:** Loredana Zocchi, Stephanie C. Wu, Claudia A. Benavente

**Affiliations:** ^1^Department of Pharmaceutical Sciences, University of California, Irvine, CA, USA; ^2^Department of Developmental and Cell Biology, University of California, Irvine, CA, USA; ^3^Chao Family Comprehensive Cancer Center, University of California, Irvine, CA, USA

**Keywords:** retinoblastoma, HELLS, E2F, RB1

Although the role of the retinoblastoma protein (pRB) in cell cycle control has been extensively studied, druggable targets downstream of pRB remain to be identified. Retinoblastoma is the most common childhood intraocular malignancy caused by bi-allelic inactivation of the retinoblastoma gene (*RB1*). Despite recent progress in clinical outcomes of retinoblastoma, enucleation (eye removal) remains a frequent treatment for retinoblastoma and the survival rate for metastatic retinoblastoma is just over 10%. Thus, there is a pressing clinical need to determine the factors responsible for tumor progression following *RB1* inactivation in order to facilitate the development of new therapeutic strategies. Furthermore, the loss of pRB function contributes to a wide array of human cancers. A few years ago, we elucidated how tumors progress quickly following *RB1* inactivation, showing that while the retinoblastoma genome is stable – with *RB1* being the only known tumor suppressor gene mutated – multiple cancer pathways can be deregulated epigenetically [1].

HELLS (helicase, lymphoid specific; also known as LSH, ICF4, PASG, and SMARCA6) belongs to the SNF2 family of chromatin-remodeling ATPases. It remodels chromatin to allow accessibility of DNMT3A or DNMT3B to DNA in order to support de *novo* DNA methylation and stable gene silencing during cellular differentiation [2]. We have previously identified that upregulation of HELLS following *RB1* inactivation could cause the epigenetic gene expression deregulation that results in tumorigenesis [3]. HELLS has an interesting connection to the RB/E2F pathway: the *HELLS* gene is a direct target of E2F1 [4] and HELLS protein interacts with E2F3 at several E2F target genes that control cell cycle entry [5,6]. Similar to what we observed in retinoblastoma, depletion of HELLS in glioblastoma and several carcinomas impairs tumor growth, suggesting that HELLS may contribute to the malignant progression of various tumors. We have also reported that HELLS is overexpressed in osteosarcoma; however, we found no evidence of HELLS serving as a driver of malignancy in these tumors [7]. The osteosarcoma study offered a precautionary perspective indicating that while HELLS level may reflect RB/E2F pathway inactivation, its upregulation may not always be synonymous with a critical role in tumor formation or tumor maintenance across all malignancies, and therefore should not be a warranted target for therapeutics against tumors in which HELLS overexpression is observed [7].

**Figure 1:**
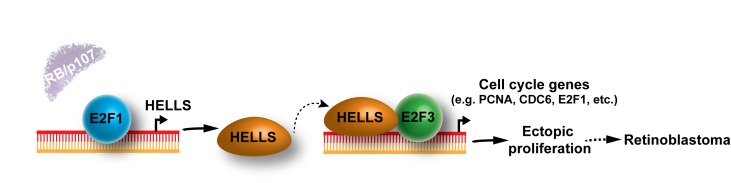
Model for HELLS function as driver of tumorigenesis in the developing retina. In the absence of the RB family (RB and p107), E2F1 drives Hells transcription, causing HELLS overexpression. HELLS protein can then function as an E2F3 transcriptional co-activator, resulting in the expression of several genes that stimulate G1/S-transition and cell proliferation that promote retinoblastoma.

In our latest study, we examined the role of HELLS in normal retina development and tumorigenesis using novel *Hells* conditional knockout mouse models. Unlike previous reports showing impaired self-renewal and maintenance in neural progenitor cells during development [8], our results indicated that *Hells*-null retinal progenitor cells (RPCs) divide, undergo cell-fate specification, and give rise to normally laminated and fully functional retinae. In spite of the dispensable role of HELLS during retinal development, genetic ablation of *Hells* in a genetically engineered mouse model of retinoblastoma led to a drastic increase in survival and decrease in morbidity compared to littermate controls [6]. Intriguingly, despite the role of HELLS in facultative heterochromatin formation, we found no global alterations in DNA methylation and only mild changes in chromatin structure variations upon Hells genetic ablation in the retinoblastoma-prone retina and tumors. Transcriptomic analyses did reveal a significant downregulation in the expression of cell cycle genes upon *Hells* genetic ablation. Interestingly, loss of Hells did not alter proliferation in retinal progenitor cells – as reflected by the normal functioning *Hells*-null retina –, yet drastically reduced cellular proliferation in the RB-null retina (i.e. initiated cells). The observations in this study have a remarkable similarity with those on the E2F family. Activator E2Fs, particularly E2F1 and E2F3, control the transcription of genes required for DNA replication and proliferation of quiescent cells but are not required for normal progenitor division in several tissues, including the developing retina [9]. However, activator E2Fs are essential for ectopic proliferation in differentiating RB-null cells such that removing E2F1 or E2F3 completely block retinoblastoma formation [3,10,11]. Thus, HELLS appear to be a critical effector of the E2F-dependent ectopic proliferation observed in the RB-null retina. We propose that in the absence of the RB family, the resulting E2F1 transcriptional derepression drives the expression of *Hells*. Consequently, HELLS can act as a transcriptional co-activator of E2F3 (also transcriptionally derepressed in the absence of RB), stimulating the expression of pro-proliferative genes (e.g. *Pcna*, *E2f1*, *Cdc6* and *Mcm4*) and retinoblastoma formation (Figure 1).

Many limitations still exist when utilizing current methods for the treatment of retinoblastoma. The significant increase in survival observed in *Hells* knockout mouse models of retinoblastoma make HELLS an interesting candidate for the treatment of retinoblastoma. HELLS is nonessential for retina development and its expression is absent in fully differentiated retina. These unique characteristics may open up a new avenue for the ocular delivery of HELLS inhibitors which holds potential in reducing tumor burden with low risk of retinal toxicities.
